# Ingestion of Bean Leaves Reduces Metabolic Complications and Restores Intestinal Integrity in C57BL/6 Mice with Obesity Induced by a High-Fat and High-Fructose Diet

**DOI:** 10.3390/nu16030367

**Published:** 2024-01-26

**Authors:** Perla Viridiana Ocampo-Anguiano, Laura Lizeth Victoria-Ruiz, Rosalía Reynoso-Camacho, Andrea Margarita Olvera-Ramírez, Nuria Elizabeth Rocha-Guzmán, Minerva Ramos-Gómez, Santiaga Marisela Ahumada-Solórzano

**Affiliations:** 1 Research and Postgraduate Department in Food Science, School of Chemistry, Autonomous University of Queretaro, Centro Universitario, Cerro de las Campanas S/N, Queretaro 76010, Mexico; pocampo129@alumnos.uaq.mx (P.V.O.-A.); lvictoria15@alumnos.uaq.mx (L.L.V.-R.); rosalia.reynoso@uaq.mx (R.R.-C.); 2 Interdisciplinary Research in Biomedicine, Faculty of Natural Sciences, Autonomous University of Queretaro, Campus Juriquilla, Av. de las Ciencias S/N, Queretaro 76230, Mexico; 3 Department of Veterinary Medicine, Faculty of Natural Sciences, Autonomous University of Queretaro, Campus Juriquilla, Av. de las Ciencias S/N, Queretaro 76230, Mexico; andrea.olvera@uaq.mx; 4 Research Group on Functional Foods and Nutraceuticals, Department of Chemical and Biochemical Engineering, TecNM/Instituto Tecnológico de Durango, Durango 34080, Mexico; nrocha@itdurango.edu.mx

**Keywords:** bean leaves, obesity treatment, intestinal integrity

## Abstract

Consumption of foods with fiber and compounds can promote gastrointestinal health and reduce obesity complications. Therefore, treatment with common bean leaves (BL) against obesity was evaluated in mice with a high-fat and high-fructose diet (HFFD) for 14 weeks. The bromatological and phytochemical characterization of BL were determined. Afterwards, the animals were supplemented with BL (10%) or a standard diet (SD) as a strategy to encourage a healthy diet for 12 additional weeks. Changes in body composition, lipid profile, and intestinal integrity were analyzed. The characterization of BL stood out for its content of 27.2% dietary fiber, total phenolics (475.04 mg/100 g), and saponins (2.2 mg/100 g). The visceral adipose tissue (VAT) decreased in the BL group by 52% compared to the HFFD group. Additionally, triglyceride levels were 23% lower in the BL consumption group compared to the HFFD group. The improvement in lipid profile was attributed to the 1.77-fold higher fecal lipid excretion in the BL consumption group compared to the HFFD group and the inhibition of pancreatic lipase by 29%. Furthermore, BL supplementation reduced the serum levels of IL-6 (4.4-fold) and FITC–dextran by 50% compared with those in the HFFD group. Metabolic endotoxemia was inhibited after BL supplementation (−33%) compared to the HFFD group. BL consumption as a treatment in obese mice reduces adipose tissue accumulation and improves the lipid profile. Furthermore, we report for the first time that BL consumption improves intestinal integrity.

## 1. Introduction

Obesity is a pathology characterized by the excess of adipose tissue and the presence of metabolic alterations, such as dyslipidemia, hyperglycemia, and insulin resistance [1]. One of the main causes of obesity is excessive consumption of fat and sugars [2]. In this sense, it is also known that excessive consumption of fructose and saturated fats modifies the synthesis of metabolites by the intestinal microbiota in the colon, which is related to systemic inflammation and alteration of intestinal integrity [1,3]. 

On the other hand, high lipid intake stimulates the synthesis of pro-inflammatory metabolites, such as indoxyl sulfuric, arachidonic, and stearic acids in the colon, which promote intestinal permeability by inhibiting the function of tight junction proteins. This inhibition promotes intestinal permeability and allows the passage of bacteria-derived toxins, such as lipopolysaccharides (LPS), from the intestinal lumen into the systemic circulation, reaching organs, such as the liver and brain, and promoting inflammation characterized, in part, by an increase in proinflammatory cytokines, such as IL-6 and IL-2 [3,4]. 

Previous studies carried out in rodents have shown that the type and quantity of macronutrients (carbohydrates, proteins, and lipids) in the diet can modify the intestinal microenvironment. Thus, the energy intake, mainly from saturated fats together with carbohydrates, especially fructose, through the diet stimulates cell proliferation, the length of the intestinal villi, and the depth of the crypts, increasing the absorption surface and, therefore, body weight gain. Meanwhile, insufficient energy intake can promote villous atrophy and a decrease in their length, further complicating nutrient absorption [4,5,6]. In addition, a relationship has been suggested between the consumption of fats and sugars with the development of alterations in intestinal integrity that promote intestinal permeability and metabolic endotoxemia [7]. 

On the other hand, there are dietary patterns that have been related to optimum health. In this regards, the WHO recommends the daily intake of 400 g of vegetables, including legumes as part of a healthy diet, since they are the main source of fiber and phytochemical compounds into the diet; in addition, they provide vitamins and minerals [8]. 

Beans are one of the most widely produced crops in Mexico. From the harvest of the seeds that are part of the basic diet of the population, the leaves that are mostly obtained are waste. The common bean (*Phaseolus vulgaris* L.) is one of the plants with the highest consumption and production in Mexico; However, most of the leaves are discarded from the harvest and only the seeds that are part of the diet and consumption of the population are rescued and marketed [9]. In Mexico, some rural populations consume this part of the plant, which is classified as one of the “quelites” or edible plants [10]. These quelites contain 24.20% of crude protein and 24.10% of total dietary fiber, as well as a minimum contribution of lipids [11]. Likewise, bean leaves are a source of iron (1.65 mg/g) and phytochemical compounds, such as total phenols (2 459 mg/100 g), soluble phenols (474 mg/100 g), and flavonoids (949 mg/100 g) [11,12]. 

The nutraceutical properties of its leaves have scarcely been investigated. Until now, it has been reported that the consumption of common bean leaves prevents the development of obesity in rats fed with HFFD by reducing weight gain (12%), abdominal fat accumulation (10%), and early insulin resistance (6%). Interestingly, the improvement in metabolic alterations is related to the significantly higher concentration of short-chain fatty acids (SCFAs, 54%) in the cecal content [11]. In another study, supplementation with bean leaves of the Eugenia variety for 13 weeks prevented hepatic lipotoxicity in rats fed HFFD [13]. Therefore, these results led us to suggest that a strategy to reduce obesity is to improve intestinal integrity through bean leaves consumption.

Bean leaves have been recommended for the prevention of obesity. However, its effect as a treatment and relationship with the improvement in intestinal integrity is unknown. Therefore, the main objective of this study was to determine the effect of bean leaves ingestion as a treatment on metabolic complications and intestinal integrity in C57BL/6 mice with obesity induced by a high-fat and high-fructose diet.

## 2. Materials and Methods

### 2.1. Bean Leaves

The *Phaseolus vulgaris* L. variety Eugenia plant was harvested in June 2019 by sowing at the Amazcala experimental campus, Autonomous University of Queretaro (Faculty of Natural Sciences, UAQ). The bean plants were grown in the open field and kept free of pesticides. After harvesting, green leaves (6–15 cm long and 3–11 cm wide) were selected, washed with distilled water, and dried in a convection oven at 45 °C up to constant weight. The dried bean leaves (BLs) were ground, sieved to 0.2 mm (Thomas-Wiley mill model 4), and stored at −80 °C.

### 2.2. Bromathological and Phytochemical Characterization of the Common Bean Leaves

#### 2.2.1. Bromatological Characterization of the Common Bean Leaves

To determine the humidity of the BL powder, 2 g of the sample was weighed in porcelain crucibles, previously placed at constant weight in a drying oven at 65 °C. The BL sample was dried to constant weight, then removed from the oven, placed in a desiccator for 15 min, then weighed again, and the difference was calculated. Ash content was determined by calcination (AOAC 942.05). The protein was analyzed by the total nitrogen macro-Kjeldahl method (AOAC 2001). Lipids were determined by the ethereal extraction method (AOAC 920.39). Total dietary fiber was obtained by an enzymatic method using a kit (Total Dietary Fiber Assay Kit, Sigma-Aldrich, St. Louis, MO, USA) with the modifications reported by Heredia-Ortega et al. [14]. All techniques were performed in triplicate on dry matter.

#### 2.2.2. Extraction of Phenolic Compounds

The sample (5 g of dried BL powder) was extracted with 10 mL of 70:30 methanol: water and sonicated three times for 30 s, with a resting of 15 s between cycles. The sample was centrifuged at 10,000× *g* for 10 min at 4 °C, and the supernatant was recovered and concentrated by using a rotary vacuum evaporator to evaporate the methanol [15]. The extract was dissolved in 1 mL of sterile distilled water and lyophilized at −60 °C [16]. For the determination of anthocyanins, the extracted sample (0.5 g) was dissolved in 4 mL of acidified ethanol (85 mL of 95% ethanol + 15 mL of 1.0 N HCl), mixed for 2 min and the pH was adjusted to 1.0 with HCl. Subsequently, the sample was shaken for 30 min, centrifuged at 10,000× *g* for 20 min, and stored at −80 °C until analysis.

#### 2.2.3. Determination of Total Phenols

The quantification of total phenols was performed by the Folin–Ciocalteu assay [17]. In a 96-well plate, 12.5 µL of the acetone extract plus 50 µL of water were placed; 32 µL of 1 N Folin–Ciocalteu reagent was added, and the oxidation reaction was neutralized with 156 µL of 7% sodium carbonate. The sample was incubated in the dark for 2 h at room temperature and the absorbance was measured at 760 nm in a plate reader (Varioskan Flash Multimode Reader, Thermo-Scientific, Waltham, MA, USA). The total phenol content was calculated from a calibration curve using gallic acid as a standard. The results are expressed in µg gallic acid equivalents/g dry sample (µg GAE/g dry sample).

#### 2.2.4. Determination of Total Flavonoids

The quantification was carried out as described by Oomah et al. [18]. For this, 50 µL of the acetone extract was placed in a 96-well plate and 180 µL of methanol was added, followed by 20 µL of a 1% solution of 2-aminoethyldiphenylborate in methanol. The absorbance was measured at 404 nm in a plate reader. The total flavonoid content was calculated from a calibration curve using rutin as a standard, and the results are expressed as µg of rutin equivalents/g of dry sample (µg RE/g of dry sample).

#### 2.2.5. Quantification of Condensed Tannins

Quantification of condensed tannins was carried out by the vanillin test described by Deshpande and Cheryan, modified for a microplate. A total of 50 µL of the acetone extract was placed in a 96-well plate and 200 µL of a freshly prepared 0.5% vanillin mixture (1% vanillin in methanol and 8% HCl in methanol in a 1:1 ratio). In addition, a blank was prepared by adding 50 µL of the extract and 200 µL of 4% HCl. The absorbance was measured in a plate reader at 492 nm. To calculate the concentrations of condensed tannins, a calibration curve was obtained with (+)-catechin (0–0.2 mg/mL). The results are expressed as mg (+)-catechin equivalents/g dry sample (mg ECAT/g dry sample) [19].

#### 2.2.6. Quantification of Total Saponins

BL powder (1 g) was weighed, and 10 mL of 80% methanol was added. The methanol was removed by rotary evaporation under vacuum conditions. A total of 1 mL of distilled water and 200 µL of acetone were added to the extract, which was centrifuged at 2500× *g* for 15 min to obtain the methanol extract. Subsequently, 0.1 mL of the methanol extract was placed and incubated with 1 mL of 72% sulfuric acid and 0.8% vanillin in ethanol. The mixture was incubated in a water bath at 60 °C for 20 min and subsequently cooled with an ice bath. The absorbance was measured at 544 nm in a plate reader, and a saponin standard (0.012–0.36 mg/mL) was used to determine the amount of saponins in the extract [20].

#### 2.2.7. Identification and Quantification of Phenolic and Flavonoids in Bean Leaves by UPLC-ESI-MS/MS

From the extracts previously obtained, the samples were resuspended in 200 μL of methanol, filtered (0.45 μm PTFE), and then resuspended at a concentration of 10 mg of extract/mL. Subsequently, 2 µL of samples were injected into a C18 reverse-phase column (Acquity UPLCr BEH C18 1.7 μm, 2.1 mm × 100 mm) at a flow rate of 0.25 mL/min. Spectrometric conditions included a capillary voltage of 2.15 kV, cone of 30 V, source output of 60 V, source temperature of 150 °C, desolvation temperature of 400 °C, a cone gas flow of 150 L/h, collision gas flow of 0.15 mL/h, and nebulization gas flow of 7.00 bar. The collision energy used in MS mode was 2.00, and in MS/MS mode was 20.00. A mixture of formic acid 7.5 mM was used as a phase A and acetonitrile was used as phase B at a flow rate of 0.25 mL/min. The following elution gradient was used: 0 min, 97% A, 3% B; 1.23 min, 91% A, 9% B; 3.82 min, 84% A, 16% B; 11.40 min, 50% A, 50% B; 13.24 min, 97% A, 3% B; 15 min, 98% A, 2% B. The results are expressed in μg/g of dry sample [15].

### 2.3. Experimental Design

#### 2.3.1. Experimental Animals

For the obesity induction phase, 72 8-week-old male C57BL/6 mice (25 to 30 g) were purchased from the Bioterio Animal of the Institute of Neurobiology (INB-UNAM, Campus Juriquilla, QRO). The total number of animals was calculated from the proposed formula that considers the prevalence of the disease to be studied (obesity) and the margin of error related to the statistical power (0.09) [21]. Animals were housed by placing 4 mice per box in standard boxes (dimensions: 19 × 29 × 12 cm), with solid continuous floors and walls, removable lids, and sterile shaving litter (1.5 cm high). The animals were kept at temperature control (22 ± 3 °C) and relative humidity (60 ± 5%) conditions under a regulated 12-hour light/dark cycle. Food and water were administered ad libitum. The cleaning of the cages, the bottles, and the feeders was carried out every third day. The experiments on animals were performed according to the Animal Care and Use protocol (NOM-062-ZOO-1999) approved by the Ethics Committee of the Autonomous University of Queretaro (Project identification code CBQ22/114, approved 21 September 2022). C57BL/6 male mice were randomly assigned into 6 experimental groups (n = 12; via the app https://es.piliapp.com/random/list/, accessed on 16 May 2022) as follows: (1) and (2) standard diet (SD), (3) and (4) high-fat and high-fructose diet (HFFD), (5) HFFD diet + bean leaves (BL consumption), and (6) HFFD diet followed by SD standard diet (change to healthy diet) to simulate a change to the recommended diet.

#### 2.3.2. Design of Diets for Experimental Animals

The SD diet (Rodent Lab Chow 5001) is formulated for the adequate nutrition and energy consumption of rodents (3.36 Kcal/g, 28.5% proteins, 13.5% lipids, and 58% carbohydrates). The BL diet was prepared by adding the BL powder to the HFFD group (10% *w*:*w*), thus simulating the recommended daily intake of vegetables per day. At the beginning of study, the animals had one week of adaptation to the powdered food base.

#### 2.3.3. Energy and Nutritional Content of Diets

The energy and the nutritional composition of the experimental diets are shown in Table 1. The diets corresponding to the HFFD and BL consumption groups were designed to be isocaloric. To keep protein intake similar between both diets (isoprotein diets), calcium caseinate was added. Additionally, to promote the absorption of dietary lipids, 0.15% sodium cholate was added [11].

#### 2.3.4. Experimental Design

The mice in groups 1 and 2 were fed the SD diet, while groups 3–6 were fed the HFFD diet. Changes in weight gain, food intake, and visceral circumferences were monitored weekly. After 14 weeks, the body weight of the HFFD-fed animals increased by ≥10% compared to that of the SD group, and thus was set as the obesity induction phase, according to the indicator of obesity reported in the literature [22]. Therefore, the animals in groups 1 (SD, n = 12) and 3 (HFFD, n = 12) were sacrificed; their large intestine was removed and washed to confirm alterations in intestinal integrity. On the other hand, the animals in the rest of the groups (2, 4–6) were supplemented according to the assigned treatments shown in Table 2 for an additional 12 weeks.

### 2.4. Changes in Body Composition from the Consumption of Bean Leaves and the Change to a Healthy Diet

#### 2.4.1. Body Composition

To evaluate the distribution of the body fat, the thoracic and abdominal circumferences were measured, and the abdominal circumference/thoracic circumference ratio was determined in the week previous to the euthanasia at the 14-week obesity-induced period and at the end of the experiment (26 weeks). 

#### 2.4.2. Quantification of Adipose Tissue

To determine the distribution of body fat, the animals were subjected to magnetic resonance imaging (Service Unit/Laboratory of the National Magnetic Resonance Imaging Laboratory, LANIREN INB-UNAM, Campus Juriquilla, QRO) in the last week before euthanasia, using isoflurane 2% as an inhalation anesthetic. The images were analyzed with the ITK-SNAP program version 3.8.0 (University of Pennsylvania, Philadelphia, PA, USA), and the results were expressed as the volume in mm^3^.

### 2.5. Role of Bean Leaves Consumption and the Change to a Healthy Diet in Lipid Metabolism

#### 2.5.1. Determination of the Content of Total Cholesterol and Triglycerides in Serum

From the serum of the different groups obtained from the euthanasia, the concentrations of cholesterol and triglycerides were analyzed with the corresponding enzymatic-colorimetric kits (Spinreact, Barcelona, Spain). The results were expressed as mg/dL.

#### 2.5.2. Total Lipids and Triglycerides in Feces

Feces were collected over 24 h in the last week of the treatment phase and stored at −80 °C. A total of 200 mg of feces was homogenized with 2 mL of 0.9% saline and shaken for 1 min, then 2 mL of chloroform–methanol (2:1 *v*/*v*) was added and the samples were shaken for 1 min. Subsequently, the samples were centrifuged at 10,000× *g* for 10 min, and the lower phase was recovered and evaporated under vacuum for 24 h. The difference in initial and final weight was determined, and the results were expressed as mg of total lipids per g of feces [23]. Additionally, triglycerides in the feces lipid extract were determined using an enzymatic colorimetric kit (Spinreact, Barcelona, Spain). The results were expressed as mg of triglycerides per g of total lipids in feces.

#### 2.5.3. Pancreatic Lipase Activity

To demonstrate the inhibition of pancreatic lipase activity by bean leaves, a sample of bean leaves powder was mixed in a water-soluble extract, following the method proposed by Pérez Ramírez et al. [24]. Briefly, 10 mg of porcine pancreatic lipase was dissolved in 1 mL of distilled water, centrifuged at 1600× *g* for 5 min, and the supernatant was mixed with different concentrations (0.1–1 mg/mL) of the bean leaf sample; samples of tetrahydrolipstatin dissolved in dimethyl sulfoxide (positive control) or dimethyl sulfoxide alone (negative control) were also included. To each sample, 400 μL of p-nitrophenyl laureate was added, and after 2 h of incubation, the oxidized glycerol product was measured at 570 nm. The results were expressed as the percentage of inhibition.

### 2.6. Euthanasia 

The euthanasia procedure in both phases (at 14 and 26 weeks) was performed by two methods, according to the subsequent analyses. In this regard, half of the animals in each group were sacrificed by administering CO_2_ by inhalation with a flow rate of 20% of the chamber volume per minute, for 60 s, and blood was subsequently obtained by cardiac puncture. Afterward, the animals were dissected, and the gastrointestinal tract was removed and washed with saline solution (0.9%). The other half of the animals (n = 6) were sacrificed by intraperitoneal administration of pentobarbital (120 mg/kg) dissolved in saline solution and subsequent cardiac puncture to draw blood. Blood samples were collected in yellow vacutainer tubes. Serum was obtained by centrifugation at 3000× *g* for 20 min and stored in aliquots at −20 °C until analyses. The cleansed intestines were placed in a 10% formaldehyde solution for paraffin embedding and the tissues were fixed on slides for the histology morphometry analysis.

### 2.7. Impact of Bean Leaves Consumption and Healthy Diet Change on the Intestinal Integrity and Metabolic Endotoxemia

#### 2.7.1. Intestinal Permeability by the FITC–Dextran Assay

The intestinal permeability was assessed by the in vivo FITC–dextran permeability assay [25] a week before the euthanasia endpoints. After a 12-hour fast, the animals were administered a dose of FITC–dextran (Sigma-Aldrich, St. Louis, MO, USA) (600 mg/kg body weight) dissolved in sterile phosphate-buffered saline (Sigma-Aldrich, St. Louis, MO) by oral cannulation. Then, 2.5 h after FITC–dextran administration, approximately 200 µL of blood was drawn via the femoral vein and centrifuged for 15 min at 3500× *g* at 4 °C; the plasma was placed in new tubes and stored at −80 °C for later analysis. The plasma FITC–dextran concentrations were assessed in a plate reader with an excitation wavelength of 490 nm and an emission wavelength of 520 nm [26]. The results are expressed in µg/mL of FITC–dextran in plasma. 

#### 2.7.2. Histopathological and Morphometric Analysis of the Jejunum and Proximal Colon

Sections of the jejunum and the proximal colon tissues were fixed in Carnoy’s solution (ethanol:chloroform:acetic acid, 6:3:1) and stained with hematoxylin and eosin to determine the extent of damage due to HFFD consumption. For this, 3 fields per section were observed in 3 sections of each mouse sample under a microscope (Velab, VE-BC3PLUS, MEX), and images were captured. The evaluation of histological damage parameters was performed by using a mouse histological guide to determine the extent of damage and the level of inflammation in crypts and villi of the jejunum and the colon tissues [27].

#### 2.7.3. Determination of LPS and IL-6 Levels in Serum

To evaluate the effect of BL consumption on metabolic endotoxemia, serum concentrations of the LPS (Abbexa LDT, Cambridge, UK) and the proinflammatory interleukin 6 (IL-6) (Express Biotech International, Frederick, MD, USA) were measured by the ELISA techniques using the corresponding kits and following the manufacturer’s instructions. The results were expressed as ng/mL for LPS and pg/mL for IL-6.

### 2.8. Statistical Analysis

The results of bean leaf composition, changes in body composition, cholesterol and triglycerides, food and water consumption, intestinal permeability test, and the serum levels of LPS and IL-6 were expressed as the means ± standard error (SEM). Quantification of phenolics acids and flavonoids in bean leaves by UPLC-ESI-MS/MS were expressed as the means ± standard deviation (SD). The chi-square test was used to analyze the histopathological changes in the colon and the jejunum portions. Changes in body composition, lipid profile, and intestinal integrity parameters were associated with the different diets using Pearson correlation. Analysis of variance (ANOVA) was used to determine significant differences among groups followed by a post hoc Tukey’s test (*p* ≤ 0.05). The statistical program GraphPad Prism 8 (1995–2022 GraphPad Software, Boston, MA, USA) was used for the graphs, and the SPSS Statistical Software version 18.0 (IBM, Mexico City, Mexico) was used for the analyses.

## 3. Results

### 3.1. Quantification of Dietary Fiber and Phytochemical Compounds

Bean leaf composition included 26.91% of protein, 27.2% of dietary fiber, and a minimal amount of lipids. On the other hand, the phytochemical characterization showed a total phenolic content of 475.04 ± 25.47 mg GAE/100 g, flavonoid content of 243.53 ± 98.40 mg EC/100 g, and a tannin content of 1.38 ± 0.17 mg EC/100 g; the total saponin content was 217.08 ± 35.69 mg/100 g. With the identification of phenolic acids and flavonoids, it was found that the predominant compounds in bean leaves are coumaric acid (1283 μg/g), caftaric acid (207 μg/g), 4-hydroxybenzoic acid (106 μg/g), quinic acid (97 μg/g), and ferulic acid derivative (95 μg/g); the predominant flavonoids are quercetin 3-O-ß-glucuronide (6892 μg/g), rutin (971 μg/g), and quercetin 3-O-glucoside (73 μg/g) (Table 3).

### 3.2. Effect of Bean Leaves on Energy Intake

During the 14-week obesity induction phase, the daily food intake (g/day) and water consumption were not affected by the type of diet; however, the energy density of the HFFD significantly increased the energy consumption of the HFFD 14-week group by 41% compared to that of the SD 14-week group. Nevertheless, no difference in the energy intake was observed between the HFFD 14-week and the HFFD 26-week groups. Unexpectedly, the energy intake of the SD 26-week group was 18% lower than the energy intake of the SD 14-week group. On the other hand, the HFFD 26-week and HFFD + BL supplementation groups had an increase in energy intake by 89.6 and 63.3%, respectively, compared to the SD 26-week group. As expected, the change to the healthy diet (SD) decreased the energy intake by 32% when compared to the HFFD 26-week group (Table 4). 

### 3.3. Treatment with Bean Leaves and Its Effect on Body Composition

#### 3.3.1. Body Weight and the Increase in the Abdominal Circumference

During the obesity induction phase, the body weight and the abdominal circumference of the animals in the HFFD 14-week group increased significantly by 23.4 and 26%, respectively, compared to those of the SD 14-week group, confirming the development of obesity. In the treatment phase, the abdominal circumference and the body weight of the animals in the HFFD 26-week group were 25.5 and 6.6% higher, respectively, compared to those of the SD 26-week group. Meanwhile, no significant differences were observed between the circumferences of the animals of the BL consumption group and the HFFD 26-week group. Although no significant decrease in body weight was observed, the abdominal circumference was significantly reduced by 18.7% by the change to a healthy diet compared to that of the HFFD 26-week group (Table 5).

#### 3.3.2. Determination of Volume Adipose Tissue

**Figure 1:**
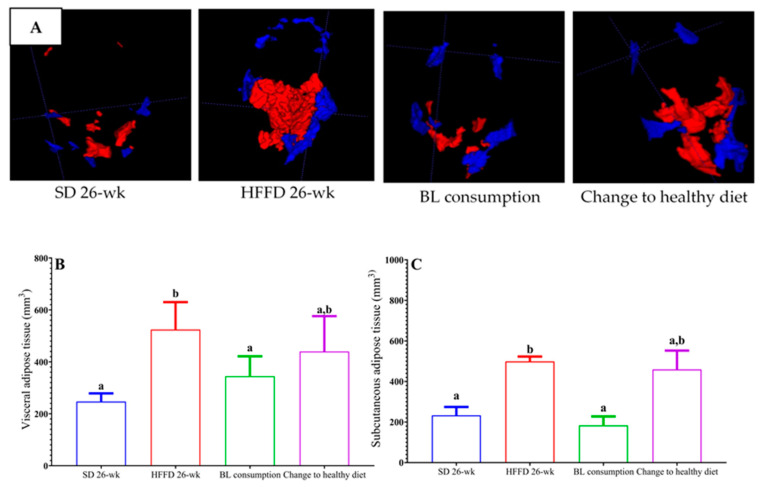
Effect of bean leaves consumption and change to healthy diet on adipose tissue in mice with induced obesity. (**A**) Total adipose tissue (images from magnetic resonance analysis); visceral adipose tissue (VAT, red) and subcutaneous adipose tissues (SCAT, blue); (**B**) visceral adipose tissue (VAT) volume; (**C**) subcutaneous adipose tissue (SCAT) volume. Data are presented as mean values ± SEM (n ≤ 3). Different letters indicate significant differences (*p* < 0.05). BL consumption = HFFD + 10% bean leaves, change to a healthy diet = SD after obesity induction phase, HFFD = high-fat and high-fructose diet, SD = standard diet.

As expected, the visceral adipose tissue (VAT) and subcutaneous adipose tissue (SCAT) of the animals in the SD 26-week group remained 56% and 48% lower, respectively, compared to those values of the animals fed the HFFD 26-week diet (Figure 1A). Interestingly, the consumption of bean leaves reduced the values of VAT by 52% and SCAT by 57%, compared to the VAT and SCAT values of the animals fed the HFFD 26-week diet. Unexpectedly, although the final body weight and the abdominal circumference significantly decreased in the animals with the change to a healthy diet, no effect on adipose tissue was observed (Figure 1B,C). 

### 3.4. Impact of the Consumption of Different Diets on the Lipid Profile

#### 3.4.1. Serum Cholesterol and Triglycerides Concentration

In the obesity induction phase, the levels of serum triglycerides and cholesterol in the animals of the 14-week HFFD group was 1.75 and 0.33 times higher, respectively, than those in the animals of the 14-week SD group (*p* < 0.05). As expected, the serum cholesterol and triglyceride concentrations were significantly higher in the HFFD 26-week group (120 mg/dL and 141 mg/dL, respectively) compared to those of the SD 26-week group (98 mg/dL and, 99 mg/dL). Interestingly, serum triglyceride levels decreased significantly by 19% in the animals of the BL consumption group, as well as in the animals fed a healthy diet by 21%, compared to the values of the HFFD 26-week group (*p* < 0.05) (Table 5).

#### 3.4.2. Lipid Excretion

**Figure 2:**
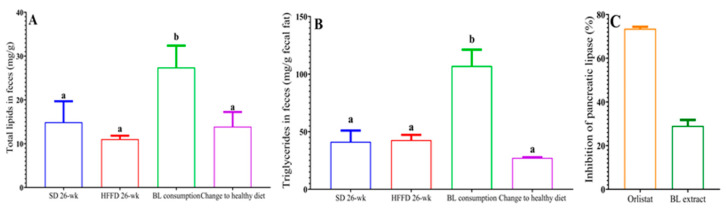
Effect of bean leaves consumption and change to healthy diet on (**A**) total lipids in feces, (**B**) triglycerides in feces, and (**C**) inhibition of pancreatic lipase. Data are presented as mean values ± SEM (n ≤ 3). Different letters indicate significant differences (*p* < 0.05). BL consumption = HFFD + 10% bean leaves, change to a healthy diet = SD after obesity induction phase, HFFD = high-fat and high-fructose diet, SD = standard diet. Orlistat = positive control of enzyme inhibition. BL extract = bean leaves methanolic extract.

Figure 2A shows the total lipids in the feces of the different experimental groups. According to the diet composition and the energy intake, the lipid content of both groups fed the standard diet was similar. However, although the HFFD group consumed more dietary fat, its lipid content was slightly lower compared to that of the SD and healthy diet groups. Interestingly, bean leaves consumption increased total lipid content by 1.74-fold compared to that of the HFFD 26-week group (*p* < 0.05). Regarding triglycerides in feces, a similar trend was observed, being significantly higher in animals fed with BL (107 mg/g) compared to animals fed with HFFD (41 mg/g) (Figure 2B). 

#### 3.4.3. Inhibition of Pancreatic Lipase In Vitro

To determine whether the decrease in adipose tissue accumulation and the serum triglyceride concentration, as well as the increase in fecal triglyceride concentration was due to lower absorption of dietary fat, the inhibition of the pancreatic lipase in vitro assay was performed. Overall, these results can be partially explained by the inhibition of this enzyme, since it was shown that the methanolic extract of BL inhibited the activity of pancreatic lipase by 29% (Figure 2C). 

### 3.5. Changes in Morphometry and Histopathology in the Intestine and colon

#### 3.5.1. Relative Weight and Length of the Small Intestine and Colon

Since the digestion and absorption of nutrients takes place in the gastrointestinal tract, we considered that changes in the small intestine and the colon could exacerbate the complications of obesity, as well as the development of inflammation. Both the small intestine and the colon lengths decreased by 19% and 27%, respectively, in the HFFD 14-week group as compared to those of the SD 14-week group, probably as an inflammatory indicator. On the other hand, the length of the small intestine after the consumption of bean leaves and the change to a healthy diet was similar to those of the HFFD 26-week and SD 26-week groups. Unexpectedly, a 23.3% decrease in the length of the colon was observed when switching to a healthy diet compared to the length of the colon of animals in the SD 26-week group (Table 6).

Similarly, the consumption of the HFFD 26-week diet decreased the relative weight of the small intestine and colon tissues by 18 and 33%, respectively, compared to those of the group fed the SD 26-week diet. As expected, the change to a healthy diet recuperated the relative weight of the colon by 23%, compared to that of animals fed with HFFD 26-week; however, no significant difference was found between the BL consumption group and HFFD 26-week group (Table 7).

#### 3.5.2. Histological Change in Intestine and Colon

**Figure 3:**
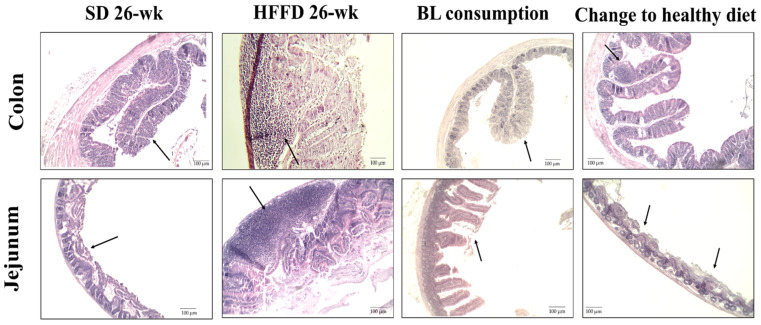
Effect of bean leaves consumption on the histopathology of the jejunum and the colon (n ≤ 5). Micrographs of colon and jejunum tissue fragments were stained with H&E and analyzed with the 10× objective lens. BL consumption = HFFD + 10% bean leaves, HFFD = high-fat and high-fructose diet, Change to a healthy diet = consumption of SD in animals with obesity, SD = standard diet. The black arrows indicate the altered structures identified by histopathological analysis.

Figure 3 shows the representative micrographs and histological characteristics of the jejunum and colon portions of the animals of the different experimental groups. In the HFFD 26-week group, 50% of the colon samples showed lymphoid hyperplasia and 25% developed villous atrophy, while in the jejunum, 50% of the analyzed structures showed infiltration of lymphoid tissue and the 25% had lymphoid hyperplasia (*p* < 0.05). Furthermore, the consumption of bean leaves improved the altered histological structures of the jejunum by 75% and the colon by 80%. Unexpectedly, 80% of the structures of the jejunum and 60% of the structures of the colon showed villous atrophy in the histological structures of the animals in the change to a healthy diet group. Likewise, infiltration of lymphoid tissue was observed in 20% of the samples analyzed from the jejunum and the colon (*p* < 0.05). As expected, the small intestine and the colon tissues of the animals fed with SD 26-week presented normal structures.

### 3.6. Intestinal Permeability and Metabolic Endotoxemia

#### 3.6.1. Intestinal Permeability

**Figure 4:**
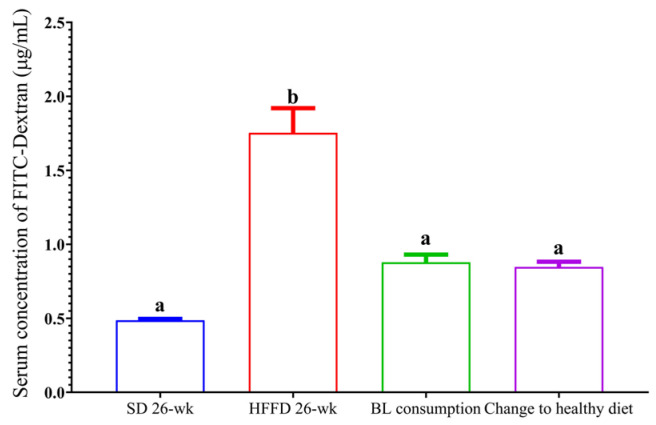
Effect of bean leaves consumption and change to a healthy diet on intestinal permeability. Data are presented as mean values ± SEM (n ≤ 3). Different letters indicate significant differences (*p* < 0.05). BL consumption = HFFD + bean leaves, change to a healthy diet = SD after obesity induction phase, HFFD = high-fat and high-fructose diet, SD = standard diet.

One of the main characteristics of the altered intestinal integrity is the increase in the permeability of the small intestine. In this regard, the serum concentration of FITC–dextran in the HFFD 26-week group increased 4-fold compared with the serum concentration of FITC–dextran in the SD 26-week group (*p* < 0.05). The intestinal permeability of the animals that consumed BL or changed to a healthy diet decreased by 50 and 55%, respectively, compared with that of the HFFD 26-week group (Figure 4).

#### 3.6.2. Metabolic Endotoxemia and Inflammation

**Figure 5:**
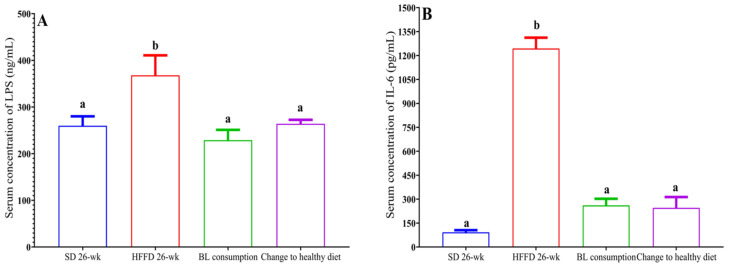
Effect of bean leaves on metabolic endotoxemia and inflammation. (**A**) LPS concentration and (**B**) IL-6 concentration. Data are presented as mean values ± SEM (n ≤ 3). Different letters (treatment period) indicate significant differences (*p* < 0.05). BL consumption = HFFD + 10% bean leaves, change to healthy diet = SD after obesity induction phase, HFFD = high-fat and high-fructose diet, SD = standard diet.

In this investigation, the serum LPS concentration in the HFFD 26-week group was 41.6% higher (*p* < 0.05) compared with that in the SD 26-week group. On the other hand, animals supplemented with bean leaves had a decrease in serum LPS concentration (60.7%), as well as animals with a change in feeding style (39.33%, *p* < 0.05) (Figure 5A). Additionally, the serum IL-6 concentration in the HFFD 26-week group was 2.3 times higher compared with the serum IL-6 concentration in the SD 26-week group (*p* < 0.05). Furthermore, animals that consumed BL and changed to a healthy diet had a significantly lower 4.5- and 3.19-fold serum IL-6 concentration, respectively, compared to the HFFD 26-week group (Figure 5B). 

#### 3.6.3. Intake of Dietary Fiber and Total Phenols

The HFFD 26-week group had a lower intake of total dietary fiber and insoluble fiber of 4.5 and 3.56 times, respectively, compared to the intake of the SD 26-week group. However, supplementation with BL increased the consumption of both total dietary fiber and insoluble fiber by 3.30 and 3.16 times, respectively, compared to those of the group that only consumed the HFFD 26-week diet. Also, the supplementation with 10% of bean leaves increased the dietary total phenolic content by 52% compared to the intake of total phenolic content of the HFFD 26-week diet (Table 8).

## 4. Discussion

Bean leaves are a food source of bioactive compounds, and in this study we confirmed that bean leaves contain dietary fiber, mainly insoluble fiber, and are a source of phenols, flavonoids, and tannins. Additionally, we report for the first time the saponin content in bean leaves. This is also relevant, since the consumption of saponins has been related to the decrease in adipose tissue and lipid profile [28]. The biotransformation of bioactive compounds from plants generates metabolites with potentially beneficial effects related to the maintenance of intestinal integrity [29]. In this sense, Becerril-Campos (2022) demonstrated that in rats with HFFD-induced obesity, 10% bean leaves prevented the development of obesity and increased the total concentration of SCFAs in the cecal contents [11]. The concentration of flavonoids in BL was similar to that reported in a previous study in the pinto villa variety [12]. 

Among the phenolic compounds the coumaric acid was the most abundant. In this regard, the administration of coumaric acid (10 mg/kg) in mice fed a high-fat diet was shown to decrease the amount of adipose tissue, the adipocyte size, and the serum leptin concentrations [30]. According to this, the proposed mechanism of action of coumaric acid is based on the inhibition of the expression of genes in white adipose tissue and the increase in the hepatic oxidation of fatty acids [30]. Among the flavonoids identified in BL, quercetin and kaempferol have shown to reduce the metabolic alterations of obesity in mice with high-fat diet. In addition, the supplementation of quercetin (0.05%) into a high-fat diet was able to downregulate the transcription of microglia proinflammatory cytokines in mice [31]. Meanwhile, the administration of kaempferol at doses between 10 to 200 mg/kg body weight can reduce body weight gain, the serum concentrations of glucose, cholesterol, and triglycerides, and the accumulation of adipose tissue in murine models induced with high-fat diets [32,33]. 

According to the nutritional composition of the most commonly consumed quelites in Mexico, such as *Amarantus hybridus* L. (17.5 to 20.3% of proteins and 2.6% of crude fiber), *Chenopodium berlandieri* (4% of protein in fresh weight and flavonoids 3935 mg/g of extract), and *Portulaca oleracea* (total phenols 9.12 mg/g and total flavonoids 1.44 mg/g in dry weight) [34], bean leaves have proved to be a competitive quelite alternative to be included in the daily diet.

In this study, the presence of tannins was also reported, and this is of major importance since tannins are hydrolyzed into gallic acid, pyrogallol, and phloroglucinol, which are precursors to acetate and butyrate. Also, urolithins are one of the main metabolites derived from tannins. More important, both urolithin A and urolithin B have been associated with the prevention of obesity in rats fed a high-fat diet by reducing lipid accumulation in the liver and by increasing lipid fecal excretion [35,36]. Overall, these results suggest the intake of bean leaves rich in fiber, phenolic compounds, saponins and tannins could be part of a healthy diet.

Animal models with obesity induced with diets rich in fat and fructose have been successfully reported to evaluate various intervention strategies. In this sense, the induction of obesity was demonstrated with the ingestion of a diet with fructose (20%) and lard (40%) for 14 weeks, characterized by a 10% overweight state with respect to the body weight of the animals fed with SD. Likewise, animals fed the HFFD showed greater abdominal circumference, greater accumulation of adipose tissue, and increased serum levels of cholesterol, and triglycerides when compared to the SD group. In addition, average food consumption (g/day) was positively correlated with energy consumption (r = 0.749). These changes were attributed to the excess of fat that provides greater energy density which promotes body weight gain (r = 0.290), as well as increased VAT (r = 0.457) and SCAT (r = 0.473) volumes. Meanwhile, it has been shown that the addition of fructose into the diet can exacerbate the metabolic alterations of obesity [37]. In previous studies, it has been confirmed that high-fat diets favor the development of obesity in a short time, while persistent intake of fructose favors the development of non-alcoholic fatty liver [38,39]. Therefore, in this study, the combination of lard and fructose in a high-calorie diet aggravated the metabolic complications.

Although we did not observe a decrease in body weight, the accumulation of VAT and SCAT, as well as the serum triglycerides was inhibited by BL consumption. Meanwhile, a change to a healthy diet reduced the abdominal circumference and the serum triglyceride concentrations. Increased abdominal circumference has been considered as an independent predictor of morbidity, associated with a greater accumulation of VAT [40]. In addition, the increase in adipose tissue can promote chronic low-grade inflammation, insulin resistance, and cardiovascular risk [41]. Interestingly, the reduction in adipose tissue due to BL consumption can be attributed to the contribution of phenolic compounds (r = −0.451). Similarly, it was previously reported that the consumption of bean leaves in rats on an obesogenic diet prevents weight gain and the amount of adipose tissue without decreasing energy consumption [11,13].

In this study, the body weight was negatively correlated with total phenol consumption (r = −0.306) and subcutaneous adipose tissue (SCAT, r = −0.452). Interestingly, it was shown that despite not decreasing body weight, BL consumption lowered adipose tissue accumulation and serum triglyceride concentrations in animals fed the HFFD. It is relevant for future research to evaluate changes in other body compartments, such as muscle mass, water content, and bone tissue to explain the lack of effect in body weight.

The increase in serum triglycerides and their ectopic accumulation is considered a risk factor for the development of diabetes and cardiovascular diseases [42]. In this study, both the intake of BL and the change to a healthy diet decreased the serum triglyceride concentrations that were formerly increased by the HFFD. Several studies have evaluated the preventive effect of consuming cooked bean seeds from different varieties (60% of the diet) and a bean pod extract (300 mg/kg body weight) by decreasing serum triglycerides in obesogenic models, which was combined with a protective effect on the development of diabetes and cardiovascular diseases [43,44]. In this study, we also demonstrated the beneficial effect of BL consumption as a treatment on hypertriglyceridemia. These outcomes could be due in part to the increase in the excretion of lipids and triglycerides in feces partially promoted by the inhibition of pancreatic lipase. These results are of great relevance, since pancreatic lipase is released into the small intestine to break down fats, along with bile salts, to promote their absorption [45]. Previous studies have found that treatments that have the ability to inhibit the activity of pancreatic lipase at levels similar to those reported in this study (29%), have effects in reducing obesity alterations [45,46]. 

As part of the effect of BL treatment on intestinal integrity, changes in the length and weight of the small intestine and the colon were also evaluated. Previous studies have reported that diets high in fat (greater than 30%) and sugar (greater than 20%) promote several alterations in the intestinal integrity [4,47]. Similarly, in this study, the small intestine and the colon of animals fed the HFFD after 26 weeks had a lower relative weight, which was not recovered by BL consumption or the change to a healthy diet. According to the literature, mice fed a high-fat diet showed a decrease in the intestinal length and weight, probably associated with intestinal inflammation that could exacerbate the intestinal permeability [25]. In addition, histopathological changes in regions of the small intestine and the colon related to alterations in intestinal integrity were also analyzed. In the jejunum of the HFFD-fed group, increased lymphocytic infiltration and hyperplasia were observed in parallel with a decrease in colonic length and an increase in circulating IL-6. Meanwhile, treatment with 10% BL restored the villi and no lymphocyte infiltration was observed. According to the literature, these results could be related to a lesser intestinal inflammation [48]. Interestingly, 75% of the jejunum portions and 80% of the colon portions of animals consuming 10% BL showed no histopathological damage, confirming the beneficial effect of BL consumption against intestinal inflammation. The histological alterations in the colon and the jejunum induced by the consumption of HFFD for 14 weeks were restored with the consumption of 10% BL for 12 weeks, protecting against potential intestinal inflammation.

In this study, the increase in serum FITC–dextran concentration associated with intestinal permeability was demonstrated. Interestingly, the change to a healthy diet and the consumption of BL reduced the serum levels of LPS and IL-6. According to the levels of metabolic endotoxemia, HFFD consumption was found to increase serum concentrations of LPS and IL-6 (r = 0.98 and r = 0.99, respectively). In contrast, changing to a healthy diet was negatively correlated with serum LPS and IL-6 concentrations (r = −0.885 and r = −0.996, respectively). Furthermore, BL consumption decreased serum LPS concentrations and was also negatively correlated with serum IL-6 concentration (r = −0.806). It should be noted that in this study the serum concentration of IL-6 decreased 4.4 times with the consumption of bean leaves, which is related to a less systemic inflammation [39].

Metabolic endotoxemia increases due to alterations in intestinal integrity that allow bacterial translocation, and this has been linked to an increase in low-grade systemic inflammation [49]. Supplementation with microbiota-accessible carbohydrates (15%) for 15 weeks has been reported to improve intestinal eubiosis by decreasing metabolic endotoxemia caused by a high-fat and low-fiber diet in mice [25]. It has also been reported that the inclusion of foods rich in fiber, such as cereals, vegetables and various legumes, into the diet has been related to the improvement in the intestinal microbiota and a decrease in systemic inflammation characterized by lower concentrations of LPS and IL-6 in circulation [38,50]. These results suggest that the consumption of bean leaves not only reduces the complications of obesity induced by a HFFD, but also reduces both the intestinal permeability and the metabolic endotoxemia.

In addition to the gastrointestinal tract promoting the digestion and absorption of nutrients, it also protects against pathogenic infections and exerts an immune function. On the contrary, an unhealthy diet high in fat and sugar decreases microbial diversity, causing leaky gut and chronic inflammation. Therefore, a nutritionally balanced diet is essential to maintain a healthy intestinal microbiome, the intestinal barrier integrity, the immunological tolerance and the normal intestinal physiology [51].

Finally, in this study, the effect of consuming whole bean leaves was evaluated, which implies the contribution of all the bioactive compounds they contain. Therefore, we cannot attribute the beneficial effects here observed to any specific compound. According to the literature, compounds previously reported or identified in this study, including coumaric acid, kaempferol, and saponins, can reduce adipose tissue accumulation and improve lipid profile [28,29,31], while quercetin consumption can participate in the inhibition of proinflammatory cytokines, which consequently reduces inflammation [32]. On the other hand, it has been proven that urolithins derived from gallic acid, one of the main metabolites of the intestinal biotransformation of tannins, can increase lipid excretion in rodents fed high-fat diets [10,34]. Furthermore, the microbial metabolites from the dietary fiber and phytochemical compounds can improve intestinal health by increasing the production of SCFAs, which was previously reported through the consumption of bean leaves [11]. Therefore, the reduction in the metabolic complications and the improvement in the intestinal integrity observed in this study can be attributed to the set of compounds present in bean leaves. 

We have demonstrated the role of consuming bean leaves as an effective treatment against obesity and the decrease in the intestinal permeability and the metabolic endotoxemia. However, it will be necessary to investigate the composition of the intestinal microbiota and their microbial metabolites to elucidate their association with the reductions in obesity and inflammatory markers.

## 5. Conclusions

Bean leaves, known as “quelites”, are low-cost and easily accessible edible plants that provide fiber and phenolic compounds that can be part of a healthy diet. In addition, bean leaves consumption can be used as a strategy for the treatment of obesity and the improvement of the intestinal integrity. Bean leaves ingestion treatment decreased the accumulation of adipose tissue and the increase in serum triglycerides induced by HFFD consumption by inhibiting the absorption of dietary lipids and protecting mice from the development of dyslipidemias. Furthermore, here we report for the first time that bean leaves consumption improves intestinal integrity by reducing permeability, metabolic endotoxemia, and inflammation. Future research could help to identify the main compounds contained in bean leaves associated with the benefits observed in this research, as well as to elucidate the mechanism of action regarding the improvement in intestinal integrity, such as the regulation of the secretion of mucins and the tight junction proteins. It is also of major relevance the identification of microbial metabolites derived from fiber and phenolic compounds and their association with the beneficial effects demonstrated after BL consumption.

## Figures and Tables

**Table 1:** Experimental groups nutritional and phenolic content of diets.

			Total Carbohydrates		Total Lipids				
	Energy (kcal/g)	Proteins (%)	(%)	Of Which Fructose (%)	(%)	Of Which Lard (%)	Crude Fiber (g/100 g)	Total Dietary Fiber (g/100 g)	Total Phenolic Compounds (mg/g)
SD	3.40	28.54	58.15	0.00	13.43	0.00	5.10	36.92	2.53
HFFD	4.70	14.28	40.90	19.98	44.81	40.17	3.07	17.31	1.55
BL consumption	4.70	13.79	41.20	20.26	45.02	40.73	3.16	21.00	3.26

Abbreviations: BL = bean leaves, HFFD = high-fat and high-fructose diet, SD = standard diet.

**Table 2:** Groups and diets of the experimental treatment phase after obesity induction.

Group	Diet
SD 26-week	Standard diet by 26 weeks
HFFD 26-week	HFFD by 26 weeks
BL consumption	HFFD + 10% BL by 12 weeks
Change healthy diet	Standard diet by 12 week

Abbreviations: BL = bean leaves consumption, HFFD = high-fat and high-fructose diet, SD = standard diet.

**Table 3:** Quantification of phenolics acids and flavonoids in bean leaves by UPLC-ESI-MS/MS.

Compounds	µg/g
Phenolic acids	
Quinic acid	97.44 ± 7.08
Caffeic acid (derivative) *	57.14 ± 3.95
Cinnamic acid (derivative) *	72.41 ± 13.00
Protocatechoic acid	8.31 ± 2.17
Caftaric acid	207.21 ± 22.20
4-hydroxybenzoic acid	106.59 ± 2.18
Coumaric acid (derivative) *	1283.81 ± 110.87
Ferulic acid (derivative) *	95.53 ± 11.82
2-hydroxybenzoic acid	96.76 ± 10.79
Benzoic acid	49.55 ± 0.54
Flavonoids	
Rutin	971.18 ± 73.19
Quercetin 3-O-ß-glucuronide	6892.20 ± 99.42
Quercetin 3-O-glucoside	73.05 ± 7.53
Kaempferol 3-O-glucoside	11.24 ± 0.10
Naringin	1.01 ± 0.36
Naringenin	2.29 ± 0.61

The results are presented as the mean and standard deviation (n = 2). * Constituents were identified in relation to major transition time. The content is expressed as the amount in µg/g of extract. Compounds identified as derivatives correspond to compounds that have different retention times and have the main compound from which they are derived in their base structure.

**Table 4:** Influence of the addition of bean leaves and the change to a healthy diet on food and water consumption in mice with induced obesity.

	Obesity Induction Phase		Experimental Treatment Phase			
	SD 14-Week	HFFD 14-Week	SD 26-Week	HFFD 26-Week	BL Consumption	Change to Healthy Diet
Food intake (g/day)	4.59 ± 0.50	4.88 ± 0.30	3.79 ± 0.40 ^a^	5.13 ± 0.80 ^a^	4.54 ± 0.60 ^a^	5.09 ± 0.50 ^a^
Energy intake (Kcal/day)	15.75 ± 1.50	22.94 ± 1.30 *	12.89 ± 1.50 ^a^	23.48 ± 3.80 ^b^	20.80 ± 2.70 ^b^	17.11 ± 1.50 ^a^
Water intake (mL/day)	4.62 ± 0.50	4.92 ± 0.40	5.05 ± 0.40 ^a^	5.61 ± 0.50 ^a^	4.90 ± 0.70 ^a^	6.10 ± 0.80 ^a^

Data are presented as mean values ± SEM (n ≤ 6). * SD vs. HFFD (obesity induction phase, *p* < 0.05). Different letters in the same row (treatment phase) indicate significant differences (*p* < 0.05). BL consumption = HFFD + 10% bean leaves, change to healthy diet = SD after obesity induction phase, HFFD = high-fat and high-fructose diet, SD = standard diet.

**Table 5:** Effect of bean leaves consumption and change to a healthy diet on the body parameters and lipid profile of mice with induced obesity.

	Obesity Induction Phase		Experimental Treatment Phase			
	SD 14-Week	HFFD 14-Week	SD 26-Week	HFFD 26-Week	BL Consumption	Change to Healthy Diet
Body weight (g)	25.63 ± 0.57	33.47 ± 0.72 *	27.96 ± 0.65 ^a^	30.50 ± 0.52 ^b^	28.09 ± 0.84 ^ab^	29.47 ± 0.42 ^a^
Abdominal circumference (cm)	7.50 ± 0.46	10.40 ± 0.43 *	7.79 ± 0.29 ^a^	8.33 ± 0.28 ^a^	8.50 ± 0.39 ^a^	6.83 ± 0.17 ^b^
Thoracic circumference (cm)	7.13 ± 0.43	9.2 ± 0.73	7.36 ± 0.26 ^a^	8.17 ± 0.25 ^a^	8.50 ± 0.55 ^a^	7.33 ± 0.33 ^a^
Serum cholesterol (mg/dL)	92.60 ± 8.60	122.80 ± 11.90 *	98.30 ± 0.90 ^a^	119.70 ± 3.40 ^b^	114.40 ± 3.50 ^b^	104.90 ± 4.20 ^b^
Serum triglycerides (mg/dL)	77.70 ± 7.60	137.30 ± 17.50 *	99.50 ± 3.90 ^a^	141.10 ± 4.20 ^b^	110.70 ± 7.30 ^a^	106.80 ± 7.70 ^a^

Data are presented as mean values ± SEM (n ≤ 6). * SD vs. HFFD (obesity induction phase, *p* < 0.05). Different letters in the same row (treatment phase) indicate significant differences (*p* < 0.05). BL consumption = HFFD + 10% bean leaves, change to a healthy diet = SD after obesity induction phase, HFFD = high-fat and high-fructose diet, SD = standard diet.

**Table 6:** Effect of bean leaves consumption and change to a healthy diet on the small intestine and the colon lengths as an indicator of intestinal inflammation.

	Obesity Induction Phase		Experimental Treatment Phase			
Length (cm)	SD 14-Week	HFFD 14-Week	SD 26-Week	HFFD 26-Week	BL Consumption	Change to Healthy Diet
Small intestine	44.00 ± 2.91	36.63 ± 1.58 *	39.85 ± 1.72 ^a^	34.31 ± 0.97 ^a^	37.95 ± 1.04 ^a^	37.24 ± 1.67 ^a^
Total colon	8.86 ± 0.50	6.98 ± 0.50	7.28 ± 0.45 ^a^	6.58 ± 0.52 ^a^	7.41 ± 0.48 ^a^	5.43 ± 0.53 ^b^

Data are presented as mean values ± SEM (n ≤ 6). * SD vs. HFFD (obesity induction phase, *p* < 0.05). Different letters (treatment period) indicate significant differences (*p* < 0.05). BL consumption = HFFD + 10% bean leaves, change to a healthy diet = SD after obesity induction phase, HFFD = high-fat and high-fructose diet, SD = standard diet.

**Table 7:** Effect of bean leaves consumption and change to a healthy diet on the relative organ weight after treatment.

Percentage (%)	SD 26-Week	HFFD 26-Week	BL Consumption	Change to Healthy Diet
Colon	1.82 ± 0.06 ^a^	1.23 ± 0.03 ^b^	1.24 ± 0.02 ^b^	1.31 ± 0.09 ^b^
Small intestine	7.06 ± 0.28 ^a^	5.77 ± 0.27 ^b^	6.25 ± 0.14 ^ab^	6.09 ± 0.14 ^b^
Liver	4.81 ± 0.14 ^a^	5.93 ± 0.28 ^ab^	5.58 ± 0.59 ^a^	4.68 ± 0.21 ^ac^

Data are presented as mean values ± SEM (n ≤ 6). Different letters (treatment phase) indicate significant differences (*p* < 0.05). BL consumption = HFFD + 10% bean leaves, change to a healthy diet = SD after obesity-induced phase, HFFD = high-fat and high-fructose diet, SD = standard diet.

**Table 8:** The consumption of bean leaves and the change to a healthy diet increases the fiber intake through the diet.

Intake (g/day)	SD 26-Week	HFFD 26-Week	BL Consumption	Change to Healthy Diet
Soluble fiber	0.21 ± 0.02 ^a^	0.08 ± 0.01 ^b^	0.24 ± 0.03 ^a^	0.33 ± 0.04 ^a^
Insoluble fiber	0.81 ± 0.08 ^a^	0.23 ± 0.02 ^b^	0.70 ± 0.09 ^a^	1.08 ± 0.13 ^a^
Total dietary fiber	1.20 ± 0.10 ^a^	0.27 ± 0.02 ^b^	0.94 ± 0.02 ^c^	1.40 ± 0.16 ^d^
Total phenolic compounds	9.59 ± 1.02 ^a^	7.95 ± 1.24 ^a^	14.8 ± 2.72 ^b^	12.87 ± 1.27 ^b^

Data are presented as mean values ± SEM (n ≤ 6). Different letters (treatment period) indicate significant differences (*p* < 0.05). BL consumption = HFFD + 10% bean leaves, change to a healthy diet = SD after obesity induction phase, HFFD = high-fat and high-fructose diet, SD = standard diet.

## Data Availability

The data are available by contacting the corresponding author.
